# Transcultural Adaptation and Validation to Spanish of the POQL Instrument in Children Aged 6 to 12 Years

**DOI:** 10.3390/medicina62061033

**Published:** 2026-05-26

**Authors:** Cristina De La Peña Lobato, Juan Carlos Cuevas-Gonzalez, María Verónica Cuevas-González, Alma Graciela García-Calderon, León Francisco Espinosa-Cristóbal, Simón Yobanny Reyes-López, Karla Lizette Tovar-Carrillo, Ixchel Araceli Maya-García

**Affiliations:** 1 Institute of Biomedical Sciences, Autonomous University of Ciudad Juárez, Juarez City 32315, Mexico; cristina.delapena@uacj.mx (C.D.L.P.L.); maria.cuevas@uacj.mx (M.V.C.-G.); alma.garcia@uacj.mx (A.G.G.-C.); leon.espinosa@uacj.mx (L.F.E.-C.); simon.reyes@uacj.mx (S.Y.R.-L.); karla.tovar@uacj.mx (K.L.T.-C.); 2 Faculty of Dentistry, Autonomous University of Campeche, Campeche City 24039, Mexico; ixcamaya@uacam.mx

**Keywords:** quality of life, pediatric patients, caries

## Abstract

*Background and Objectives*: Oral health is an important component of overall health, including in children, since dental caries is the most frequent oral health condition in this demographic. It affects children’s daily performance and can lead to complications ranging from moderate discomfort to highly disabling problems, which are reflected in their quality of life. Validating instruments that provide reliable information to measure how oral health impacts children’s quality of life will help prioritize the management of these problems through personalized treatments. The aim of this study was to perform transcultural adaptation and Spanish validation of a POQL instrument in children aged 6 to 12 years who attended the Pediatric Dentistry Clinic at the Universidad Autónoma de Ciudad Juárez (UACJ), and to establish an association between the presence of carious lesions and the quality of life of children. *Materials and Methods*: We conducted a validation study involving a sample of 379 children aged 6 to 12 years who were attending the Pediatric Dentistry Clinic at the Universidad Autónoma de Ciudad Juárez. The instrument, adapted into Spanish, was applied to measure oral health-related quality of life, and the clinical diagnosis of caries was established using the ICDAS II system. *Results*: The mean age of the children was 8.51 years ± 1.64; 50.4% were boys and 49.6% girls. A total of 45.9% of the children presented caries with ICDAS II codes 5 and 6, corresponding to a severe stage with advanced tooth destruction, and 52% of the children reported their perception of their oral health-related quality of life as good. In the bivariate statistical analysis, the chi-square test showed no relationship between moderate and severe ICDAS II stages and the children’s perception of their quality of life, resulting in a very low Spearman correlation. *Conclusions*: The findings suggest that this instrument may represent a reliable and valid tool for use in children aged 6 to 12 years. The observed association between different degrees of carious lesions and children’s quality of life may reflect the close relationship between oral health and important psychosocial domains, including physical, emotional, and social development, which constitute the core dimensions evaluated by the POQL instrument.

## 1. Introduction

Oral health is an important component of an individual’s overall well-being, as it allows for adequate physical and emotional development, ultimately contributing to a better quality of life. Oral health is affected by dental caries and periodontal disease, which are the main conditions present in the population. These diseases represent a global public health problem, with dental caries being the leading cause of dental consultations at any stage of life [1,2].

Oral health issues have physical, economic, and social consequences that can influence an individual’s perception of their quality of life. In this sense, one of the objectives of oral care is to promote quality of life through healthy behaviors throughout a person’s lifetime, while recognizing that it is the patient who defines their perception of quality of life, not the health professional. Over the years, different instruments have been designed to evaluate the relationship between oral health and quality of life; however, these generally focus on adults or patients with specific diseases or conditions, and most are available in English [3,4].

The Pediatric Oral Health-Related Quality of Life (POQL) instrument pays special attention to children, as they are an important target of public health measures [4]. This instrument was developed by a group of researchers at Boston University, emphasizing the importance of collecting information about children’s perspectives on their dental health. To achieve this, instruments originally designed for adults had to be adapted and validated. Implementing the POQL in dental consultations only added six minutes to the appointment while providing direct information from the child about how they perceived their oral health. This instrument has been validated in different metropolitan populations in the United States, without being limited to any specific ethnic group [5].

The instrument is composed of different domains divided into physical, emotional, social functioning, and role activity. Each domain captures a distinct dimension of the impact of oral health on the child. It is a short instrument with 16 items that can be easily answered by patients, providing important information that is not routinely collected. Understanding the quality of life of pediatric patients allows for more personalized diagnoses, improving oral health conditions in this population [6].

For this reason, and given the relevance of the topic, this study aimed to carry out a transcultural adaptation and validation of the POQL instrument in Spanish for children aged 6 to 12 years and to determine the association between the presence of carious lesions and the quality of life of children.

## 2. Materials and Methods

The study was conducted at the Pediatric Dentistry Clinic of the Universidad Autónoma de Ciudad Juárez with a total sample of 379 children aged 6 to 12 years who attended dental consultations between August 2024 and May 2025. The objective was to perform a transcultural adaptation to Spanish and validate the Pediatric Oral Health-Related Quality of Life (POQL) instrument and establish the relationship between quality of life and oral health status, specifically by measuring the presence of dental caries. This research was approved by the Ethics Committee of the Institute under resolution number CEI-2024-1-1247. The study was performed in three phases: translation of the POQL instrument, validation of the instrument, and association between the dental health status of children (ICDAS II) and quality of life, using the previously validated POQL instrument.

### 2.1. Translation of the Instrument

The POQL instrument was translated and adapted into Spanish, with items adjusted according to the study population. This process involved three experts in the field (a pediatric dentist, a researcher with experience in instrument validation, and a child psychologist.) Once the translated version was obtained (this was done by a bilingual person fluent in English and Spanish), it was sent to ten professionals with experience in providing dental care to children and who have knowledge of research (pediatric dentists with master’s degrees). These professionals work within university settings as well as in private practice and they were asked to rate the items and determine their relevance, indicating whether they were essential, useful but not essential, or unnecessary. This was done using Lawshe’s method to measure the content validity ratio.

To ensure that the items were appropriate for respondents in terms of comprehension, a pilot test was conducted with a group of 99 children aged 6 to 12 years who attended dental consultations at the UACJ Pediatric Dentistry Clinic during the study period.

### 2.2. Validation of the Instrument

The validity of the pilot test was assessed using the Kuder–Richardson 20 analysis to evaluate the internal reliability of the dichotomous items (0.82). To measure the reliability of the instrument, the test–retest method was applied, which consisted of re-administering the questionnaire to the 99 pilot participants thirty days after the first administration (a sufficient period to avoid memory bias and/or response variation); this approach allowed measurements to be collected at two different points in time. Once the results were obtained, the instrument was applied to the entire sample. Exploratory and confirmatory factor analyses were conducted to examine the structure of the instrument in the study population (EFA/CFA).

### 2.3. Association Between ICDAS and the POQL Instrument

A standardization process and calibration were carried out for caries detection diagnosis according to the criteria of the International Caries Detection and Assessment System (ICDAS II). A public health expert trained in the ICDAS method participated in the standardization process, aiming to achieve high concordance values between the two examiners. This procedure was carried out from January to July 2024 and consisted of several sessions, including theoretical standardization conducted prior to ethics committee approval and practical standardization conducted after approval on 16 May 2024. During the practical standardization phase, 30 children were examined (inter-observer kappa: 0.77 and 0.72; intra-observer kappa: 0.90 and 0.91, respectively). As part of the examination process, children with restorations were evaluated for the presence of secondary carious lesions, and in cases of multiple carious lesions, the highest score was recorded.

Children aged between 6 and 12 years whose parents or guardians signed the informed consent were included. Children older than 7 years signed an informed assent form. All procedures were explained in age-appropriate language. Parents or guardians provided information on the child’s identification record and medical history. Children individually answered the pediatric patient quality-of-life instrument, which consisted of three polytomous questions and 10 dichotomous questions. The questionnaire was read individually to each child, and responses were recorded by the researcher. The process took approximately 5 min per child.

A database was specifically designed in the statistical program SPSS version 22 and R program version 3.6 to perform data analysis, which was descriptive and bivariate.

## 3. Results

### 3.1. Translations and Validation of the Instrument

Following its translation into Spanish, the instrument was subjected to evaluation by a panel of 10 experts to determine the content validity ratio using Lawshe’s method, demonstrating acceptable and valid results for each individual item (Supplementary Material S1).

To assess the internal consistency of the instrument, a pilot study was conducted in a sample of 99 children using the Kuder–Richardson 20 (KR-20) coefficient, yielding a value of 0.82; this was indicative of good reliability. Item-level analysis showed that the exclusion of any individual item did not significantly alter the overall KR-20 coefficient, supporting the stability of the instrument.

Test–retest reliability was evaluated by administering the questionnaire to the same population on two separate occasions and calculating the intraclass correlation coefficient (ICC), which yielded a value of 0.69, indicating an acceptable level of reliability (Table 1).

Exploratory factor analysis revealed a clear four-factor structure consistent with the instrument’s theoretical framework. Confirmatory factor analysis demonstrated an excellent model fit (CFI = 0.95, TLI = 0.93, RMSEA = 0.061, SRMR = 0.041), supporting the structural validity of the scale across its physical, role, social, and emotional dimensions (Supplementary Materials S2 and S3 and Image S1).

### 3.2. Association Between ICDAS and POQL Instrument

A total sample of 379 children was obtained, of whom 191 were boys (50.4%) and 188 were girls (49.6%). Children aged 6 to 12 years were included, with a mean age of 8.51 years ±1.64. Regarding the number of siblings, 34% of participants (*n* = 131) had one sibling, 32.7% (*n* = 124) reported having two siblings, while 13.2% (*n* = 50) indicated being only children.

In relation to place of birth, most children included in the study were born in Ciudad Juárez, Chihuahua (*n* = 285, 75%). Additionally, 12% (*n* = 48) were born in El Paso, Texas, and 12.1% (*n* = 46) in other locations in the country. Of the 379 children included in the study, 207 (54.6%) attended dental consultations for general check-ups, 147 (39%) for curative treatment, and only 25 (6.6%) for preventive treatment.

Regarding accompaniment during consultations, 329 children (86.8%) were brought up by one of their parents. Twelve children (3.2%) attended with their grandparents, while the rest were accompanied by other relatives or caregivers. No statistically significant differences were observed between sex and age, place of birth, number of siblings, birth order, reason for consultation, or accompanying person, nor with any descriptive variable (Table 2).

Concerning oral hygiene habits, 175 children (46.2%) brushed their teeth twice a day, 113 (29.8%) three times a day, 88 (23.2%) once a day, while only one child (0.3%) reported not brushing at all. Four participants (1.1%) reported brushing more than three times a day.

Regarding the use of additional oral hygiene products, only 57 children (15%) reported using complementary items such as dental floss or mouthwash, while the remaining 322 (85%) used only a toothbrush and toothpaste. Most children (345, 91%) brushed their teeth autonomously, without adult supervision, while only 34 children (9%) did so under supervision. As for the time of day when brushing was performed, 156 children (41.2%) brushed in the morning and at night, 125 (33%) in the morning, afternoon, and night, 49 (12%) only in the morning, and 36 (9.5%) only at night. The rest reported brushing at other times of the day (Table 3).

Oral health status was classified specifically by measuring the presence of caries among the 379 children into four categories according to the presence of carious lesions. A total of 53 children (14%) presented good dental health, with no evidence of caries, corresponding to code 0 of the ICDAS II system. Meanwhile, 121 children (31.9%) were in the initial stage of incipient caries, with lesions classified under codes 1 and 2. Additionally, 31 children (8.2%) presented moderate-stage caries (codes 3 and 4), in which structural damage to dental tissue was already observed. Finally, 174 children (45.9%) were diagnosed with severe-stage caries, characterized by advanced tooth destruction and the presence of deep cavities, corresponding to codes 5 and 6 of the ICDAS II system (Table 4).

Regarding children’s perception of quality of life in relation to oral health, 200 participants (52%) reported a positive perception, classifying it as good. Meanwhile, 151 children (40%) indicated a moderate perception, and 28 (8%) considered their quality of life to be poor (Table 5).

Bivariate analysis using the chi-square test did not show a significant association between oral health status (moderate and severe stages according to ICDAS II) and children’s perception of their oral health-related quality of life (Table 6 and Supplementary Material S4).

Spearman’s correlation coefficient (Rho = 0.20) indicates a weak positive association between the variables.

## 4. Discussion

Studying the impact of oral health on children’s quality of life is highly relevant, as it allows healthcare professionals to prioritize the dental treatment needs of this population. The validation of the POQL instrument in children from Ciudad Juárez supports the instrument’s structure and confirms its structural validity across its four dimensions.

This study evaluated oral health and quality-of-life perception in 379 children aged 6 to 12 years using the ICDAS II caries detection system and the POQL instrument adapted to the studied population. Regarding caries detection, much of the literature uses the DMFT (decay, missing, and filled teeth) or deft indices (decayed, extracted, and filled teeth); however, ICDAS II requires greater examiner standardization. It was chosen for this study because it provides more detailed information. Overall, most children presented caries in initial and severe stages according to ICDAS II, without this affecting their perception of quality of life, which was generally reported as good.

Sociodemographic data related to sex showed no significant differences with any variable, reflecting a balanced distribution between boys and girls. Regarding dental hygiene habits, significant differences were observed between boys and girls in relation to the time of day when toothbrushing was performed (*p* = 0.048). Most children reported brushing in the morning and at night (41.16%) or in the morning, in the afternoon, and at night (32.9%), with this routine being more frequent among girls.

In terms of supervision during brushing, no significant differences were found between sexes. Notably, more than 90% of children brushed without supervision, which is a considerable point of discussion in this population. This highlights the need to promote monitoring of oral hygiene habits, especially at early ages, to establish healthy routines. As Molina-Frechero et al. emphasize, adults play a crucial role in teaching children oral hygiene habits to ensure good oral health at any age. Thus, the caregiver’s role is essential [7].

Significant differences were observed in general health perception between boys and girls (*p* = 0.04). Of the surveyed girls, 3.7% reported good health perception compared to 34.5% of boys. More than 80% of the children examined presented some degree of caries, which is a very high percentage, like that reported by Márquez Pérez et al., who found prevalences of up to 88.5% in 2020 and 2021. Although there has been a decrease in caries prevalence among Mexican children, this disease remains associated with biological, behavioral, and socioeconomic conditions [8].

In the study presented by García-Jau et al., the ICDAS II grades (both dentitions) most frequently identified were grade 2 with 75.58% (294) and grade 5 with 50.39% (87). Grades 1 and 4 were less frequent, with 11.83% and 15.42%, respectively [9]. These results coincide with our study, where codes 2 and 5 were also the most frequent, with the highest percentages. However, Peña and Zavarce reported differing results, where code 04 (dark shadow in dentin visible through wet enamel with cavitation) was present in 24.8%, and code 05 (cavity with visible dentin of less than 0.5 mm and up to 50% of the surface) in 18% [10].

The findings obtained through the ICDAS II system review show that most children present dental caries, ranging from severe stages with a result of 45.9% to 31.9% in the initial stage. No statistically significant differences were observed between boys and girls regarding the presence of caries, indicating a similar condition between these two groups.

One of the strengths of this study is the use of the ICDAS II method to determine oral health status, as it has high reproducibility and sensitivity for caries diagnosis and for detecting incipient changes in teeth, thus avoiding overlooking early carious lesions during clinical examinations. This coincides with Cerón Bastidas (2015) [2], who mentions that several studies have demonstrated that ICDAS has greater diagnostic capacity compared to the radiographic method, being considered more accurate and with excellent reproducibility. Likewise, it is attributed to detecting the earliest changes in the optical properties of enamel, which are not detectable through radiographs [2].

This study measured the relationship between oral health and quality of life through the POQL instrument, which was validated into Spanish for this research. Regarding dental health perception, 56.45% of participants rated it as “good,” “very good,” or “excellent,” while 8.7% considered it “poor.”

The results show that 51.7% of participants reported having experienced dental pain, while 48.3% had not experienced it. This finding is relevant, as dental pain is one of the main reasons for dental consultation and can significantly affect quality of life, although the results suggest that experiencing pain does not substantially affect perceived quality of life. This finding may reflect the considerable adaptive capacity of children; nevertheless, additional factors should be explored to more comprehensively explain this behavior. Additionally, 39.6% of respondents expressed concern about the condition of their teeth or mouth, compared to 60.4% who did not express such concern, indicating that a considerable percentage of individuals had worries about their oral health. No statistically significant differences were found between boys and girls. Other studies have reported that sex may influence habits, perception, and the presence of oral diseases [11].

Regarding quality-of-life perception, 52.7% of children rated it as good, while 40% rated it as moderate, with no statistically significant differences by sex. These results are relevant, as this is an opportune moment to improve patients’ oral health conditions and prevent future complications at older ages, when quality of life will indeed be affected and restorative treatments—which are more costly and therefore less accessible to much of the population—will be required.

We emphasize, based on various results, that at least at this stage of life, there is no relationship between oral conditions and quality of life, despite the participants being in moderate-to-severe stages according to the ICDAS II classification for caries diagnosis. This may be due to the average age of participants being 8.5 years, which influences their perception of their environment, as younger children perceive their surroundings differently compared to older subjects. As reported by Verdugo et al. (2002), from the age of 12 there are modifications in the way quality of life is perceived [12].

We believe it is relevant to maintain oral health in this age group to avoid future complications and ensure quality of life is not affected. However, it is important to distinguish between statistical and clinical significance, as the lack of statistically significant findings in our study does not necessarily imply the absence of clinical relevance regarding the presence of carious lesions. Pain experiences associated with dental caries may still have a meaningful impact on children’s quality of life, particularly in cases of severe pain, which can directly influence their interaction with their environment.

The main limitation of this study is its restriction to patients aged 6 to 12 years and its focus on analyzing the association between quality of life and the presence of carious lesions. An additional limitation is the lack of assessment of potential confounding variables related to oral health, such as dietary patterns, which should be considered in future research.

However, these data provide a precedent for future research including older subjects to clarify whether quality of life undergoes modifications and maintains any relationship with the patient’s oral health status.

## 5. Conclusions

It is important to emphasize that the primary objective of this study was the transcultural adaptation and Spanish validation of the POQL instrument, which is designed to assess oral health-related quality of life. The findings suggest that this instrument may represent a reliable and valid tool for use in children aged 6 to 12 years. The observed association between different degrees of carious lesions and children’s quality of life may reflect the close relationship between oral health and important psychosocial domains, including physical, emotional, and social development, which constitute the core dimensions evaluated by the POQL instrument. Overall, these findings may contribute to future research exploring the perception of quality of life in relation to various aspects of oral health.

## Figures and Tables

**Table 1:** Test–retest method, in which a good level of reliability is observed for most items.

ITEMS	Intraclass Correlation Coefficient	*p* Value	Correlation
1. How would you rate your health in general	0.70	0.002	Good
2. In general, how would you rate the health of your teeth and mouth?	0.62	0.001	Good
3. Compared to one year ago, how would you describe the health of your teeth and mouth now?	0.70	0.08	Good
4. Did you have pain because of your teeth or mouth?	0.69	0.001	Good
5. Did you have trouble eating any foods (hard/hot/cold) because of your teeth or mouth?	0.68	0.001	Good
6. Did you have trouble paying attention in school because of your teeth or mouth?	0.71	0.002	Good
7. Did you miss school because of your teeth or mouth?	0.90	0.002	Good
8. Did you not want to laugh or smile around others because of your teeth or mouth?	0.70	0.002	Good
9. Did you worry that you were not good looking to others because of your teeth or mouth?	0.70	0.028	Good
10. Were you unhappy with the way you looked because of your teeth or mouth?	0.65	0.002	Good
11. Were you angry or upset because of your teeth or mouth?	0.59	0.003	Moderate
12. Did you feel worried because of your teeth or mouth?	0.58	0.002	Moderate
13. Did you cry because of your teeth or mouth?	0.75	0.001	Good
14. In general, how would you describe your experiences with your dentist?	0.73	0.030	Good
15. When was your last visit to a dentist?	0.73	0.002	Good
16. What was the reason(s) for your last dental visit?	0.73	0.002	Good

**Table 2:** Sociodemographic characteristics by sex. General characteristics of sociodemographic data show significant differences between males and females according to the chi-square test with a significance level of *p* < 0.05.

Sociodemographic Data								
	Female		Male		Total			
	#	%	#	%	#	%	X^2^	*p*
***Age***								
**6**	28	7.3	23	6.0	51	13.4	3.84	0.837
**7**	33	8.7	31	8.1	64	16.8		
**8**	38	10.0	40	10.5	78	20.5		
**9**	36	9.4	34	8.9	70	18.4		
**10**	34	8.9	40	10.5	74	19.5		
**11**	11	2.9	17	4.4	28	7.3		
**12**	8	2.1	6	1.5	14	3.6		
***Siblings (count)***								
**0**	28	14.9	22	11.5	50	13.2	4.06	0.255
**1**	50	30.9	73	38.2	131	34.6		
**2**	68	36.2	56	29.3	124	32.7		
**3 or more**	34	18.1	40	20.9	74	19.5		
***Family birth order***								
**1**	81	43.1	82	42.9	163	43	0.944	0.624
**2**	70	37.2	78	40.8	148	39.1		
**3 or more**	37	19.7	31	16.2	68	17.9		
***Birthplace***								
**Juárez**	149	39.3	136	35.8	285	75.1	4.91	0.086
**ELP**	23	6.0	25	6.5	48	12.6		
**Other state**	16	4.2	30	7.9	46	12.1		
***Reason for visit***								
**Check-up**	104	27.4	103	27.1	207	54.6	0.674	0.714
**Preventive treatment**	14	3.6	11	2.9	25	6.5		
**Curative Treatment**	70	18.4	77	20.3	147	38.7		
***Relationship with the patient***								
**Parents**	165	43.5	164	43.2	329	86.8	4.438	0.35
**Grandparents**	5	1.3	7	1.8	12	3.1		
**Siblings**	0	0	4	1.0	4	1.0		
**Uncles/Aunts**	1	0.2	1	0.2	2	0.05		
**Others**	17	4.4	15	3.9	32	8.4		

**Table 3:** Distribution of dental hygiene habits by sex.

*Dental Hygiene Habits by Sex*								
	Female		Male		Total			
	#	%	#	%	#	%	X^2^	*p*
***Time of the day for brushing***								
**Morning, afternoon, night**	65	17.1	60	15.8	125	32.9	11.19	0.048
**Morning, night**	88	23.2	68	17.9	156	41.1		
**Morning**	18	4.7	31	8.1	49	12.9		
**Afternoon**	12	3.1	24	6.3	36	9.4		
**Night**	2	0.5	2	0.5	4	1.0		
**Other**	3	0.7	6	1.5	9	2.3		
***Supervision during brushing***								
**No**	174	45.9	171	45.1	345	91.0	1.06	0.37
**Yes**	14	3.6	20	5.2	34	8.9		
***Use of dental hygiene products***								
**Yes**	29	7.6	28	7.3	57	15.0	0.04	0.88
**No**	159	41.9	163	43	322	84.9		
***Perception of general health***								
**Good**	143	37.7	131	34.5	274	72.2	8.22	0.04
**Fair**	30	7.9	51	13.4	81	21.3		
**Poor**	11	2.9	8	2.1	19	5.0		
**Don’t know**	4	1.0	1	0.2	5	1.3		

**Table 4:** Results of data on caries levels using the ICDAS II system. Sex-based distribution of caries stages.

	Female			Male			Total			
	#	%	Age Mean	#	%	Age Mean	#	%	X^2^	*p*
***Presence of caries ICDASII***										
**Cavity-free**	31	8.1	9.3	22	5.8	9.4	53	13.9	2.71	0.43
**Initial stage**	57	15.0	8.5	64	16.8	8.9	121	31.9		
**Moderate stage**	13	3.4	9.2	18	4.7	8.3	31	8.1		
**Severe stage**	87	22.9	7.9	87	22.9	8.1	174	45.9		

**Table 5:** Sex-based distribution of dental health perception and quality of life.

	Female		Male		Total			
	#	%	#	%	#	%	X^2^	*p*
***Dental Health perception***								
**Excellent**	13	3.4	23	6.0	36	9.4	5.37	0.25
**Very good**	19	5.0	15	3.9	34	8.9		
**Good**	79	20.8	65	17.1	144	37.9		
***Fair***	61	16.0	71	18.7	132	34.8		
**Poor**	16	4.2	17	4.4	33	8.7		
***Quality of life perception***								
**Good**	95	25.0	105	27.7	200	52.7	0.8	0.67
**Moderate**	79	20.8	72	18.9	151	39.8		
***Poor***	14	3.6	14	3.6	28	7.3		

**Table 6:** Results from the Oral Health-Related Quality-of-Life instrument and ICDAS.

	Oral Health-Related Quality of Life			
	*Good Quality of Life (Score 0–3)*	*Moderate Quality of Life (Score 4–7)*	*Poor Quality of Life (Score 8–10)*	*X^2^ (p)*
	n (%)	n (%)	n (%)	
***ICDAS 0***	36 (18)	13 (8.6)	4 (14.3)	22.6 (0.01)
***ICDAS 1–2***	77 (38.5)	35 (23.2)	9 (32.1)	
***ICDAS 3–4***	15 (7.5)	13 (8.6)	3 (10.7)	
***ICDAS 5–6***	72 (36)	90 (59.6)	12 (42.9)	

## Data Availability

The original contributions presented in this study are included in the article. Further inquiries can be directed to the corresponding author(s).

## Supplementary Materials

The following supporting information can be downloaded at: https://www.mdpi.com/article/10.3390/medicina62061033/s1, Supplementary Material S1. Child self-report POQL instrument questions; Supplementary Material S2. shows a model structure in the four dimensions: physical, role, social, and emotional; Supplementary Material S3. Confirmatory factor analysis confirms a good model fit (CFI = 0.95, TLI = 0.93, RMSEA = 0.061, SRMR = 0.041), which validates the validity of the POQL instrument; Supplementary Material S4. POQL instrument results; Image S1. The observed factor structure supports the validity of the theoretical model, showing that the physical, role, social, and emotional dimensions are interrelated and contribute jointly to the construct being evaluated.

## Abbreviations

The following abbreviations are used in this manuscript:

OH	Oral health.
POQL	Pediatric Oral Health-Related Quality of Life.
ICDAS II	International Caries Detection and Assessment System.
