# Supplementary material for: Transcultural Adaptation and Validation to Spanish of the POQL Instrument in Children Aged 6 to 12 Years
Source: Medicina (Kaunas). 2026 May 26;62(6):1033. doi: 10.3390/medicina62061033 (PMC13304306; doi:10.3390/medicina62061033)

Supplementary material S1.- Child self-report POQL instrument questions

Items	CONTENT VALIDITY ESSENTIAL	USEFUL, BUT NOT ESSENTIAL	NOT NECESSARY	CVR
1.How would you rate your health in general	X			1
2. In general, how would you rate the health of your teeth and mouth?	X			1
3. Compared to one year ago, how would you describe the health of your teeth and mouth now?	X			1
4. Did you have pain because of your teeth or mouth?	X			1
5. Did you have trouble eating any foods (hard/hot/cold) because of your teeth or mouth?	X			1
6. Did you have trouble paying attention in school because of your teeth or mouth?		X		0.8
7. Did you miss school because of your teeth or mouth?				1
8. Did you not want to laugh or smile around others because of your teeth or mouth?	X			1
9. Did you worry that you were not good looking to others because of your teeth or mouth?		X		0.8
10. Were you unhappy with the way you looked because of your teeth or mouth?	X			1
11. Were you angry or upset because of your teeth or mouth?		X		0.8
12. Did you feel worried because of your teeth or mouth?		X		0.8
13. Did you cry because of your teeth or mouth?		X		0.8
14. In general, how would you describe your experiences with your dentist?		X		0.8
15. When was your last visit to a dentist?		X		0.8
16. What was the reason(s) for your last dental visit?		X		0.8

Supplementary material S2.- shows a model structure in the four dimensions: physical, role, social, and emotional.

Exploratory factor analysis EFA)		
Factor	Item	Factor loading
Physical	4. Did you have pain because of your teeth or mouth?	0.74
	5. Did you have trouble eating any foods (hard/hot/cold) because of your teeth or mouth?	0.69
Role	6. Did you have trouble paying attention in school because of your teeth or mouth?	0.63
	7. Did you miss school because of your teeth or mouth?	0.60
social	8. Did you not want to laugh or smile around others because of your teeth or mouth?	0.71
	9. Did you worry that you were not good looking to others because of your teeth or mouth?	0.76
	10. Were you unhappy with the way you looked because of your teeth or mouth?	0.80
Emotional	11. Were you angry or upset because of your teeth or mouth?	0.65
	12. Did you feel worried because of your teeth or mouth?	0.62
	13. Did you cry because of your teeth or mouth?	0.58

Supplementary material S3.- Confirmatory factor analysis confirms a good model fit (CFI = 0.95, TLI = 0.93, RMSEA = 0.061, SRMR = 0.041), which validates the validity of the POQL instrument.

EXPLORATORY CONFIRMATORY ANALYSIS (CFA)				
Factor	Item	Loading (λ)	error	R^2
Physical	4. Did you have pain because of your teeth or mouth?	0.74	0.45	0.55
	5. Did you have trouble eating any foods (hard/hot/cold) because of your teeth or mouth?	0.69	0.52	0.48
Rol	6. Did you have trouble paying attention in school because of your teeth or mouth?	0.63	0.60	0.40
	7. Did you miss school because of your teeth or mouth?	0.60	0.64	0.36
Social	8. Did you not want to laugh or smile around others because of your teeth or mouth?	0.71	0.50	0.50
	9. Did you worry that you were not good looking to others because of your teeth or mouth?	0.76	0.42	0.58
	10. Were you unhappy with the way you looked because of your teeth or mouth?	0.80	0.36	0.64
Emotional	11. Were you angry or upset because of your teeth or mouth?	0.65	0.58	0.42
	12. Did you feel worried because of your teeth or mouth?	0.62	0.62	0.38
	13. Did you cry because of your teeth or mouth?	0.58	0.66	0.34

Supplementary material S4.- POQL instrument results

**ORAL HEALTH RELATED QUALITY OF LIFE (POQL)**		
**ITEM**		**n (%)**
**1. How would you rate your health in general**	Good	274 (72.3)
	Fair	81 (21.4)
	Poor	19 (5)
	don't know	5 (1.3)
**2. In general, how would you rate the health of your teeth and mouth?**	Excellent	36 (9.5)
	Very good	34 (9)
	Good	144 (38%)
	Fair	132 (34.8)
	Poor	33 (8.7)
**3. Compared to one year ago, how would you describe the health of your teeth and mouth now?**	Excellent	30 (7.9)
	Very good	49 (12.9)
	Good	150 (42)
	Fair	104 (27.4)
	Poor	37 (9.8)
**4. Did you have pain because of your teeth or mouth?**	183 (48.3)	196(51.7)
**5. Did you have trouble eating any foods (hard/hot/cold) because of your teeth or mouth?**	212(55.9)	167(44.1)
**6. Did you have trouble paying attention in school because of your teeth or mouth?**	309(81.5)	70(18.5)
**7. Did you miss school because of your teeth or mouth?**	240 (63.3)	139 (36.7)
**8. Did you not want to laugh or smile around others because of your teeth or mouth?**	282(74,4)	97 (25.6)
**9. Did you worry that you were not good looking to others because of your teeth or mouth?**	252 (66.5)	127(33.5)
**10. Were you unhappy with the way you looked because of your teeth or mouth?**	284 (74.9)	95 (25.1)
**11. Were you angry or upset because of your teeth or mouth?**	278 (73.4)	101 (26.6)
**12. Did you feel worried because of your teeth or mouth?**	150 (39.6)	229 (60.4)
**13. Did you cry because of your teeth or mouth?**	231 (60.9)	148 (39.1)

**14. In general, how would you describe your experiences with your dentist?**		
**15. When was your last visit to a dentist?**	Fist time	64 (16.9)
	once every 6 months	117 (30.9)
	once a year	45 (11.9)
	when there is a need for treatment	104 (7.4)
	don’t know	49 (12.9)
**16. What was the reason(s) for your last dental visit?**	Regular checkups	124 (32.7)
	Preventative Care	34 (9)
	Treatment	189 (49.9)
	Don’t remember	32 (8.4)

Image S1. The observed factor structure supports the validity of the theoretical model, showing that the physical, role, social, and emotional dimensions are interrelated and contribute jointly to the construct being evaluated.

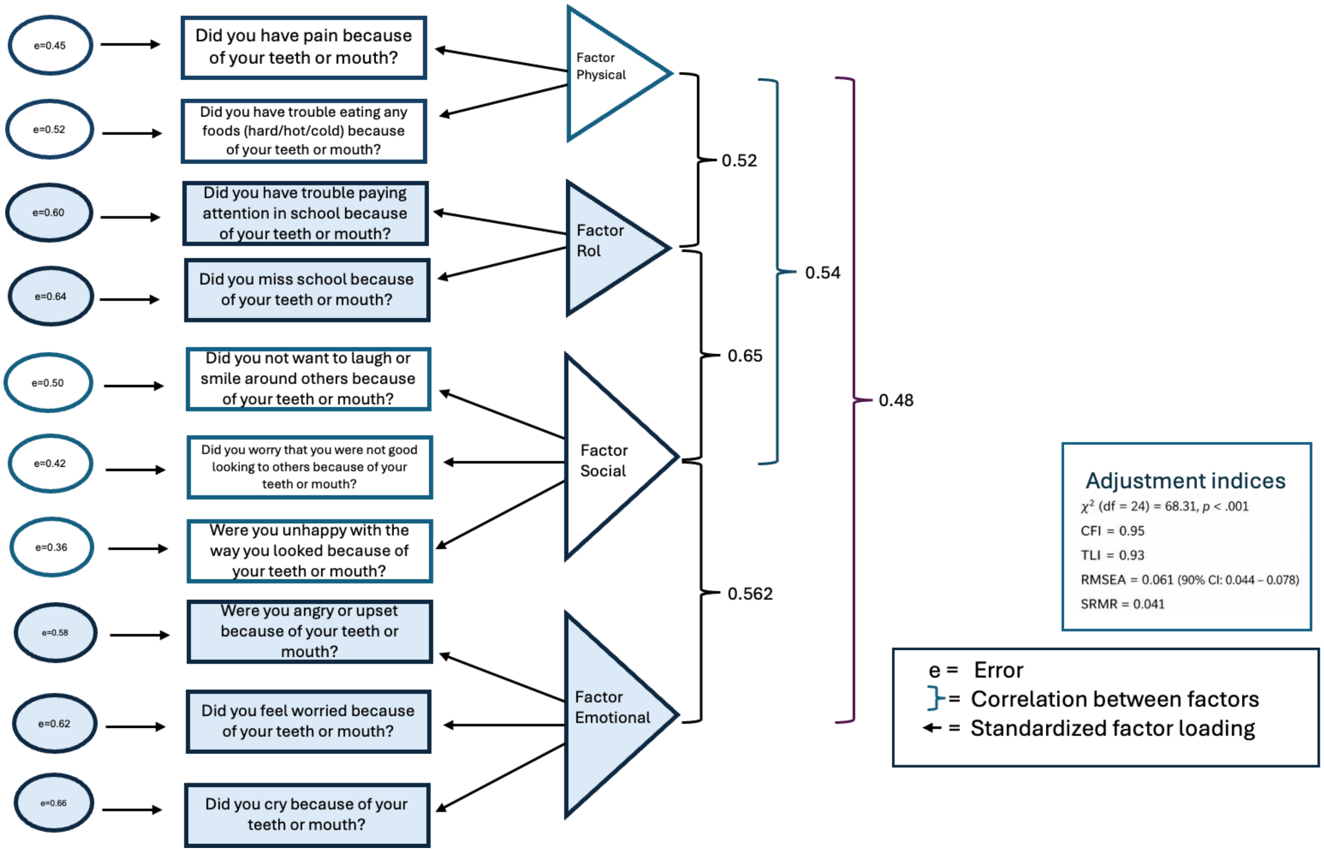

Supplement: Supplementary file 1 [file medicina-62-01033-s001.zip › medicina-4260454-supplementary.pdf]
